# Novel *Vigna subterranea* (L.) Verdc Soluble Dietary Fibre*-*Starch Nanocomposite: Functional and Antioxidant Characteristics

**DOI:** 10.17113/ftb.60.03.22.7409

**Published:** 2022-09

**Authors:** Yvonne Maphosa, Victoria Adaora Jideani, Daniel Imwansi Ikhu-Omoregbe

**Affiliations:** 1 Department of Food Science and Technology, Cape Peninsula University of Technology, P.O. Box 1906, 7535 Bellville, South Africa; 2 Department of Chemical Engineering, Cape Peninsula University of Technology, P.O. Box 1906, 7535 Bellville, South Africa

**Keywords:** bambara groundnut nanocomposite and starch, dietary fibre source, antioxidant properties, pasting properties

## Abstract

**Research background:**

Bambara groundnut (*Vigna subterranea* (L.) Verdc.) is a great source of soluble dietary fibre and starch. Bambara groundnut soluble dietary fibre is rich in bioactive compounds, namely, uronic acids (11.8%) and hydrolysable polyphenols (expressed as gallic acid equivalents (GAE) 20 mg/g), with crucial physiological and functional benefits. The industrial use of native starch is limited because of the inherent undesirable attributes that render it unstable. The aim of this study is to characterise the antioxidant, functional and physicochemical properties of Bambara groundnut starch-soluble dietary fibre nanocomposite (hereafter groundnut nanocomposite).

**Experimental approach:**

The pasting properties by rapid visco analysis, chemical composition, hydration properties, oil-binding capacity, emulsifying activity index (EAI), emulsion stability index (ESI) and antioxidant properties of Bambara groundnut starch, soluble fibre and nanocomposite were studied.

**Results and conclusions:**

The Bambara groundnut soluble dietary fibre and nanocomposite did not exhibit typical pasting properties. The nanocomposite had high mass fraction of carbohydrates (78.7%) and proteins (7.0%), low mass fraction of fat (0.8%) and had a considerable mass fraction of ash (4.9%). The Bambara groundnut starch, soluble dietary fibre and nanocomposite showed significant (p<0.001) differences in solubility. Their EAI values were 23.2, 85.7 and 90.6%, respectively, and the ESI values were 23.3, 87.1 and 87.5%, respectively. The three biopolymers differed significantly (p<0.001) in all colour characteristics: lightness (L*), redness/greenness (a*), yellowness/blueness (b*), chroma and hue angles. Their polyphenolic mass fraction, expressed as GAE, was 0.10, 6.6 and 0.46 mg/g, respectively, and their ferric reducing antioxidant power values, expressed as ascorbic acid equivalents (AAE), were 1.2, 4.8 and 1.4 μmol/g, respectively. The phenolic compounds (in mg/g): chlorogenic acid 18, monocrotaline 20, luteolin 7-O-(6''-malonylglucoside) 4 and casuarine 6-α-d-glucoside 27 were present in the dietary fibre but absent from the starch and nanocomposite. The Bambara groundnut nanocomposite possesses desirable physicochemical and antioxidant properties, making it suitable as an ingredient in various food systems.

**Novelty and scientific contribution:**

Nanocomposites have the potential to revolutionise the food industry but their study as food ingredients is very limited. Furthermore, nothing is known about the physicochemical, functional and antioxidant characteristics of Bambara groundnut nanocomposite, thus investigating these properties will address this knowledge gap.

## INTRODUCTION

The demand for innovative food products formulated with natural components has driven the constant search for new biopolymer complexes with improved properties. The increasing demand for such complexes, both by consumers and the food industry, has led to the utilisation of biopolymers from underutilised and orphan crops, such as Bambara groundnut (*1*). Starch and dietary fibre from different sources have been studied and applied in the production of various food products (*2*). Starch and starch-based composites immensely contribute to the functional properties of food products (*3*). The major functional properties of starch and dietary fibre include thickening, swelling, gelation, water absorption, water binding, foaming, emulsification and bulk density (*4*). Bambara groundnut starch and soluble dietary fibre have been reported to possess good functional properties and their complexes have improved pasting, thickening, viscoelastic and thermal properties better than starch alone (*5*).

Previous studies have reported the effect of complexing starch with other biopolymers on the functionality, antioxidant composition and overall behaviour of the formed complexes. Such studies reported on composites made from potato starch and waxy maize starch (*6*), waxy rice starch and non-waxy starch (*7*), lima bean starch and cassava starch (*8*) as well as potato starch and maize starch (*9*). It is important to study the functional and physicochemical properties of food components as they affect sensory characteristics as well as the behaviour of food during formulation, processing, storage and distribution (*1*, *2*). Knowledge of the functional and antioxidant properties of biopolymer composites enables their maximal utilisation and application in food systems for human nutrition and health (*5*).

The nanocomposite used in this study was a starch-soluble dietary fibre nanocomposite (hereafter groundnut nanocomposite), produced from the chemical complexing of Bambara groundnut soluble dietary fibre and Bambara groundnut starch. The groundnut nanocomposite was reported to have an average particle size of 74.01 nm, which qualifies it as a nanocomposite (*d*<100 nm) (*10*). The application of this nanocomposite in a food system would produce a relatively ’green’ product as all components used are natural. The objective of this study is to characterise the antioxidant, functional and physicochemical properties of the Bambara groundnut nanocomposite.

## MATERIALS AND METHODS

### Source of materials

Analytical grade chemicals from Sigma-Aldrich, Merck (Johannesburg, South Africa) were used in this study. Bambara groundnut nanocomposite (we named STASOL) is a starch-soluble dietary fibre nanocomposite made from the chemical complexing of Bambara groundnut soluble dietary fibre and its starch. Starch and soluble dietary fibre were extracted from the black-eye Bambara groundnut variety as described by Maphosa *et al.* (*10*) and the nanocomposite used in this study was produced following the method described by the same authors. In a 100-mL Schott bottle, 15 g Bambara groundnut starch, 37.5 mL deionised water, 16.5% H_2_O_2_ (*V*=120 mL) and 0.1% ascorbic acid were mixed and then incubated at 90 °C for 45 min in a temperature-controlled water bath (Ecobath, LaboTec, Midrand, South Africa). The mixture was cooled to room temperature before precipitating with 40 mL absolute ethanol dropwise with continuous agitation in a sonicator (model  W-225R; Heat Systems Ultrasonics, now Qsonica, LLC, Newtown, CT, USA) for 10 min at high speed. The mixture was then centrifuged (Jouan MR1812; Thermo Electron Corporation, Thermo Fisher Scientific, Waltham, MA, USA) at 3783×*g* for 5 min and the supernatant was discarded. To obtain regenerated Bambara groundnut starch particles and to remove H_2_O_2_ and ascorbic acid, the residue was rinsed three times with 40 mL absolute ethanol. The Bambara groundnut starch (15 g) and its soluble dietary fibre (1.95 g) were dissolved in 40 and 15 mL *φ*(ethanol,water)=0.5, respectively. The two solutions were mixed to obtain a molecular dispersion of the starch and fibre and left to react for 24 h on a magnetic stirrer (model MS-H-ProT; DLAB Scientific, Beijing, PR China), in a dark fume hood. The resulting solution was freeze-dried (VirTis Genesis 25EL pilot lyophilizer; SP Industries Inc, Warminster, PA, USA).

### Pasting properties of Bambara groundnut starch, soluble dietary fibre and starch-soluble dietary fibre nanocomposite 

Pasting properties were analysed using a Rapid Visco Analyser (RVA 4500; Perten Instruments, PerkinElmer, Brisbane, Queensland, Australia) following the method of Gulu (*1*). A 3-gram sample (14% moisture) in 25 g deionised water was subjected to a heating and cooling cycle while continuously under shear stress. The samples were held at 50 °C for 1 min and then heated from 50 to 95 °C at 6 °C/min. Thereafter, the temperature was decreased to 50 °C at 6 °C/min and held for 5 min. The pasting time, peak viscosity, pasting temperature, breakdown viscosity, cold paste viscosity and setback viscosity were obtained from the RVA curves and viscosities were expressed in Pa·s.

### Colour characteristics of Bambara groundnut starch, soluble dietary fibre and starch-soluble dietary fibre nanocomposite

Colour characteristics of all Bambara groundnut samples were determined using a spectrophotometer (model CM-5; Konica Minolta Sensing Inc, Sakai, Japan) set at standard observer 10° and D65 following a method of Murevanhema and Jideani (*11*). The spectrophotometer was calibrated with a black tile (L*=5.49, a*=7.08, b*=4.66) and a white tile (L*=93.41, a*=1.18, b*=0.75) followed by zero calibration (*4*). Enough samples to completely cover the bottom of the glass sample holders (*d*=30 mm) were used. Colour parameters were assessed using L*C*h* and CIE-L*a*b* colour space systems. Colour differences (∆*E*) among the samples were calculated using the colour difference equation:



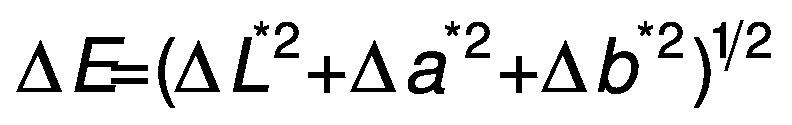



where L* is lightness, a* is redness/greenness and b* is yellowness/blueness (*12*).

### Chemical analysis of Bambara groundnut starch, soluble dietary fibre and starch-soluble dietary fibre nanocomposite

Chemical profiling was carried out according to AOAC (*13*) procedures for total fat (gas chromatography), protein (TruSpec® N; Leco Corp., St. Joseph, MI, USA), moisture (moisture analyser IR-30; Denver Instrument Arvada, CO, USA), ash (muffle furnace), minerals (inductively coupled plasma-optical emission spectrometry) and carbohydrates (calculated by difference). Energy (kJ) was calculated using the corresponding values of fat (37 kJ/g), protein (17 kJ/g) and carbohydrates (17 kJ/g) (*13*).

### Hydration properties of Bambara groundnut starch, soluble dietary fibre and starch-soluble dietary fibre nanocomposite

Water absorption capacity (WAC) and solubility index were determined according to the methods described by Lai *et al.* (*14*). To determine WAC, 0.2 g samples were weighed into centrifuge tubes and mixed with 9.8 g deionized water. The suspensions were heated at 70 °C for 10 min in a shaking water bath (Ecobath, LaboTec) and then transferred to a boiling water bath for another 10 min. The suspensions were cooled and then centrifuged at 40 000×*g* and 5 °C for 30 min (Jouan MR1812; Thermo Electron Corporation, Thermo Fisher Scientific). WAC was determined as the ratio of the mass of swollen samples after centrifugation (g) to their dry mass (g).

To determine the solubility index, the supernatant from WAC analysis was decanted into tared evaporating dishes and dried overnight at 110 °C. Solubility index was then calculated as the ratio of the mass of solids after drying (g) to the initial mass of the dry sample (g).

### Oil-binding capacity of Bambara groundnut starch, soluble dietary fibre and starch-soluble dietary fibre nanocomposite

The method of Maphosa and Jideani (*4*) was applied for the determination of the oil-binding capacities (OBC) of Bambara groundnut starch, its soluble dietary fibre and nanocomposite. Samples (1 g) were mixed with 5 g canola oil in 50-mL centrifuge tubes. The mixtures were vortexed for 30 s at 5 min intervals for 30 min, then centrifuged (Jouan MR1812; Thermo Electron Corporation, Thermo Fisher Scientific) at 1600×*g* and 23 °C for 25 min. After centrifugation, the free oil was decanted and weighed. The difference between the original mass of oil (5 g) and the mass of the decanted oil was considered as the retained oil. OBC was calculated as the mass of retained oil divided by the original mass of the sample.

### Activity and stability of Bambara groundnut starch, soluble dietary fibre and starch-soluble dietary fibre nanocomposite emulsions

Emulsifying activity index (EAI) and emulsion stability index (ESI) were determined following the methods of Chove *et al.* (*15*). Samples (1 g) were dissolved in 10 mL deionised water and homogenised (Ultra Turrax T-25 homogeniser; IKA, Staufen, Germany) at 4500×*g* for 2 min. Orange oil (10 mL) was added, the solution was homogenised at 6000×*g* for 1 min and then centrifuged again at 1200×*g* for 5 min. To calculate EAI, the emulsion volume was measured in mL of emulsified layer per entire layer in the centrifuge tube and expressed as a percentage (%). To calculate ESI, the emulsion was heated at 80 °C for 30 min. After cooling to ambient temperature, the emulsion was centrifuged at 1200×*g* for 5 min and the ESI was calculated as the height of the emulsified layer after heating divided by the height of total content before heating.

### Total phenolic content and antioxidant activity of Bambara groundnut starch, soluble dietary fibre and starch-soluble dietary fibre nanocomposite

Total phenolic compounds were determined following the method of Maphosa and Jideani (*4*). Samples (250 mg) were mixed with 10 mL of distilled water and 1 mL of H_2_SO_4_ in 14-mL centrifuge tubes and then incubated at 80 °C for 20 h before centrifugation (4000×*g*, 5 min, 21 °C). The supernatant was diluted tenfold and analysed in a 96-well plate using the Folin-Ciocalteu assay by mixing 25 µL sample with 125 µL of 0.2 M Folin-Ciocalteu reagent and 100 µL of 7.5% Na_2_CO_3_ solution. The mixtures were left to stand for 2 h in the dark. Absorbance was then measured with a spectrophotometer (Multiskan Spectrum; Thermo Fisher Scientific) at a wavelength of 765 nm using a gallic acid standard calibration curve. The results were expressed as mg of gallic acid equivalents (GAE) per g of dry extract.

Ferric reducing antioxidant power (FRAP) assay was conducted following the method of Maphosa and Jideani (*4*) with vitamin C as a standard. Samples (250 mg) were diluted tenfold and then mixed with 0.3 mL FRAP reagent (30 mL acetate buffer, 3 mL FeCl_3_, 3 mL 2,4,6-tri(2-pyridyl)-*s*-triazine (TPTZ) and 6 mL H_2_O). The mixture was poured into a 96-well plate, left to stand for 30 min and then read in a spectrophotometer (Multiskan Spectrum; Thermo Fisher Scientific) at a wavelength of 593 nm. The results were expressed as µmol of ascorbic acid equivalents per g of dry extract.

### Determination of glucose mass fraction in Bambara groundnut starch, soluble dietary fibre and starch-soluble dietary fibre nanocomposite

Samples (0.1 g) and 5 mL of 2 M trifluoroacetic acid were accurately weighed into 10-mL headspace vials and hydrolysed at 110 °C for 12 h. The mixtures were then diluted with 0.2% triethylamine and 0.2% NH_4_OH in water and with 0.2% triethylamine, 0.1% 2-propanol and 0.2% NH_4_OH in acetonitrile. The diluted samples were transferred into glass vials and analysed by ultra-performance liquid chromatography–evaporative light scattering detection (UPLC-ELSD) with a Waters Acquity BEH Amide 2.1 mm×100 mm, 1.7 µm column (Waters, Milford, MA, USA). An injection volume of 5 µL was used with a column temperature of 50 °C, seal wash of 5 min, run time of 10 min and a pressure gradient of 0-1.03421·10^8^ Pa/min.

### Phenolic profiling of Bambara groundnut starch, soluble dietary fibre and starch-soluble dietary fibre nanocomposite

Samples (2 g) and 15 mL of 50% methanol and 1% formic acid were accurately weighed into 50-mL centrifuge tubes with screw-caps, then vortexed for 1 min, followed by extraction in an ultrasonic bath for 1 h. Samples (2 mL) were then centrifuged at 8827×*g* for 5 min and the supernatants were transferred into 1.5-mL glass vials using liquid chromatography-mass spectrometry (LCMS). A Waters Synapt G2 quadrupole time-of-flight (QTOF) mass spectrometer (MS) connected to a Waters Acquity UPLC was used for high-resolution UPLC-MS analysis. The column eluate first passed through a photodiode array detector prior to flowing through the mass spectrometer, allowing concurrent collection of UV and MS spectra. Electrospray ionisation was used in negative mode with a cone voltage of 15 V, desolvation temperature of 275 °C, desolvation gas at 650 L/h, and the rest of the MS settings optimised for best resolution and sensitivity. Data were acquired by scanning from 150 to 1500 *m*/*z* in resolution mode as well as in MSE mode. In MSE mode two channels of MS data were acquired; one at low collision energy (4 V) and the other using a collision energy ramp (40-100 V) to obtain fragmentation data. Leucine enkephalin was used as a reference mass and the instrument was calibrated with sodium formate. Separation was achieved on a Waters HSS T3, 2.1 mm×100 mm, 1.7 μm column and the mobile phase was made up of 0.1% formic acid (solvent A) and acetonitrile containing 0.1% formic acid (solvent B). The used parameters were: injection volume 2 μL, flow rate 0.3 mL/min and column temperature 55 °C. The gradient began at 100% solvent A for 1 min, then went to 28% solvent B over 22 min, then changed to 40% solvent B over 50 s and a wash step of 1.5 min at 100% solvent B, and finally re-equilibrated to initial conditions for 4 min. A range of catechin standards were injected from 0.5 to 100 mg/L and used to establish a calibration curve against which compounds were quantified. Data were processed using MS-DIAL and MS-FINDER (RIKEN Center for Sustainable Resource Science, Metabolome Informatics Research Team, Kanagawa, Japan).

### Data analysis

For statistical analysis, IBM Statistical Package for the Social Science (SPSS) v. 25 was used (*16*). All experiments were carried out in triplicate. Data were expressed as mean±standard deviation. The results were subjected to multivariate analysis of variance (MANOVA) to establish differences between treatments. Duncan’s multiple range test was used to separate mean values where a significant (p≤0.05) difference existed.

## RESULTS AND DISCUSSION

### Bambara groundnut starch, soluble dietary fibre and its nanocomposite pasting properties

The pasting characteristics of Bambara groundnut starch, Bambara groundnut soluble dietary fibre and its nanocomposite are given in Table 1. Pasting is a term used to describe the changes in the structure of starch molecules after gelatinisation (*17*), such as further swelling of granules, their disruption and leaching of molecular components from them. Several studies have reported the pasting properties of Bambara groundnut starch (*1*, *2*, *5*, *17*). As expected, its soluble dietary fibre and nanocomposite did not exhibit typical pasting properties since pasting is a characteristic of starch molecules.

**Table 1:** Pasting properties (viscosity, peak time and pasting temperature) of Bambara groundnut starch (BGNS), Bambara groundnut soluble dietary fibre (BGN-SDF) and Bambara groundnut starch-fibre nanocomposite (STASOL)

Sample	*η*/(mPa∙s)					*t*_p_/min	Temperature (pasting)/°C
	**Peak**	**Trough**	**Breakdown**	**Final**	**Setback**		
BGNS	(4875±219)^a^	(1984±944)^a^	(2890±1143)^a^	(4621±500)^a^	(2637±577)^a^	(4.8±0.0)^a^	(77.9±2.0)^a^
BGN-SDF	(435±16)^b^	(412±20)^b^	(24±4)^b^	(537±9)^b^	(122±9)^b^	(7.0±0.0)^b^	(96.7±3.0)^b^
STASOL	(150±10)^c^	(143±1)^b^	(7±1)^b^	(271±9)^b^	(128±0)^b^	(6.9±0.0)^c^	(87.4±0.0)^c^

Values are mean±standard deviation. Mean values followed by different superscripts within a column differ significantly (p≤0.05). *t*_p_=time when peak viscosity is reached

#### Peak viscosity

Peak viscosity refers to the highest viscosity of starch obtained during gelatinisation (*18*). It is indicative of the strength of starch pastes and their water-holding capacity (*19*, *20*). The peak viscosities of Bambara groundnut starch, its soluble dietary fibre and nanocomposite were 4875, 435 and 150 mPa·s (Table 1), and all three differed significantly (p<0.001). Peak viscosity is an indication of structural damage due to factors such as temperature and shear, with high values indicating more extensive damage (*2*, *21*).

Bambara groundnut starch-soluble dietary fibre nanocomposite had a significantly (p<0.001) lower peak viscosity than the starch and soluble dietary fibre; therefore, it would be expected to maintain its structure at relatively high temperatures. This was confirmed by thermal studies (*10*) where the structural degradation of the nanocomposite occurred at 293.14 °C. The structural damage of the Bambara groundnut starch at a lower temperature of 77.19 °C (*10*) was due to its ability to gelatinise (*22*). Starch is composed of repeating glucose units, dietary fibre is a mixture of chemically complex non-starch polysaccharides and nanocomposite is a mixture of the starch and soluble dietary fibre, suggesting that new functional groups were introduced during the chemical grafting process (*10*). The significantly (p<0.001) reduced peak viscosity of the nanocomposite could be attributed to several factors such as the disruption of starch granules during the chemical grafting process resulting in the loss of gelatinisation ability as well as the simplicity of the starch molecule compared to the other two compounds, hence allowing it to swell unrestrictedly (*2*). Furthermore, the differences in the microstructure and morphology of Bambara groundnut starch and nanocomposite were discussed by Maphosa *et al.* (*10*), and the starch granules were reported to be spherical with smooth surfaces while the nanocomposite was irregular and polygonal. These differences were attributed to the disruption of starch bonds during chemical grafting as well as the formation of new bonds when the soluble dietary fibre was added to the nanocomposite. Since peak viscosity is also an indication of water-binding capacity of starch (*21*); the higher peak viscosity of Bambara groundnut starch means that in comparison to its soluble dietary fibre and nanocomposite, the starch granules swell more easily when heated and form a thicker paste.

Gulu (*1*) reported a similar trend in the peak viscosity of native and chemically modified starches, reporting a reduction in their peak viscosities from 4.876 to 0.435 Pa·s, respectively. Oyeyinka *et al.* (*5*) reported a lower peak viscosity of 3.293 Pa·s for Bambara groundnut starch. The difference in the reported peak viscosity could be due to the fact that starches from different Bambara groundnut varieties were studied. We used starch from the black-eye variety in this study, while Oyeyinka *et al.* (*5*) used starch from the cream variety. Lower starch peak viscosities of 1.335, 2.152 and 4.145 Pa·s were reported for wheat (*19*), barley (*23*) and potato (*24*), respectively. Differences in peak viscosities of starches can be attributed to the source of starch, differences in amylose and amylopectin contents, strength of amylose-amylose and amylose-amylopectin chain interactions as well as the molecular structures of amylose and amylopectin (*19*).

#### Trough viscosity

Trough viscosity is also known as hot paste viscosity, and it describes the rate of breakdown in viscosity to equilibrium. A low trough viscosity indicates higher paste stability. The trough viscosity of Bambara groundnut starch (1984 mPa·s) was significantly (p=0.012) higher than that of its soluble dietary fibre (412 mPa·s) and nanocomposite (143 mPa·s) (Table 1). Gulu (*1*) reported a similar trend where chemical modification of the starch using hydrogen peroxide and ascorbic acid resulted in a decrease in trough viscosity. The same author reported a trough viscosity of 0.088 Pa·s for chemically modified Bambara groundnut starch, while a trough viscosity of 143 mPa·s was reported in this study. The differences in the results indicate that grafting catechin onto the starch as was done by Gulu (*1*) resulted in the formation of a more robust complex than grafting Bambara groundnut soluble dietary fibre onto the starch to form the nanocomposite. Trough viscosity depends on temperature, degree of shear stress applied to a mixture and the nature of the material of interest. A higher trough viscosity (3.013 Pa·s) than in our study for Bambara groundnut starch was reported by Gulu (*1*). The differences in the results could be because Gulu (*1*) studied the starch from a mixture of different varieties of Bambara groundnut seeds, while in this study we used starch from the black-eye variety.

Trough viscosities of 0.560 Pa·s for wheat starch (*19*), 1.423 cPa·s for barley starch (*23*), 2.351 Pa·s for potato starch (*24*) and 5.280 Pa·s for barley starch (*25*) have been reported. These are starches commonly used in the food industry. Their trough viscosities were very high, indicating that they would breakdown when exposed to elevated temperatures and form unstable pastes. As such, more stable composites such as Bambara groundnut starch-soluble dietary fibre nanocomposite with low trough viscosity (0.412 Pa·s) would be desirable replacements. The relatively low trough viscosity of the nanocomposite suggests that it would find use in high temperature food systems such as baking and high shear processes such as those involved in the production of emulsions and dough.

#### Final viscosity

The final viscosities of Bambara groundnut starch, its soluble dietary fibre and nanocomposite were 4621, 537 and 271 mPa·s, respectively (Table 1). Final viscosity gives an indication of the ability of starch to form a gel or viscous paste after cooking and cooling (*26*). It is also used to determine starch quality and stability of the cooked starch paste in food products. According to Falade *et al.* (*21*), final viscosity plays a vital role in the rigidity and stability of the swollen granule structure. Higher final viscosities of 4.867, 4.876, 4.989 and 5.591 Pa·s were reported for rice (*27*), Bambara groundnut (*1*), quinoa (*27*) and barley starch (*25*), respectively. The lower final viscosity of the nanocomposite than of these starches was due to the loss of crystallinity during the grafting process. This was confirmed by crystallinity studies using powder X-ray diffraction (*10*) where native Bambara groundnut starch was described as crystalline in nature while its nanocomposite was amorphous. The transformation from crystalline to amorphous nature of legume starch as a result of chemical treatment has been widely reported (*22*). The lower final viscosities of the soluble dietary fibre and nanocomposite would result in the formation of more stable food systems. Therefore, these two biopolymers would make suitable alternatives to modified starch.

#### Breakdown viscosity

The breakdown viscosities of the starch, its soluble dietary fibre and nanocomposite were 2890, 24 and 7 mPa·s, respectively (Table 1). Both the soluble dietary fibre and nanocomposite had significantly (p=0.003) lower breakdown viscosities than the starch. Breakdown viscosity is an indication of the starch organisation structure and measures the susceptibility of starch granules to disintegration (*26*). A higher breakdown viscosity means the material has low heat and shear resistance (*21*). Bambara groundnut starch had the highest breakdown viscosity and therefore would have low thermal stability compared to the soluble dietary fibre and nanocomposite (*20*). This was in agreement with thermal studies (*10*). Bambara groundnut starch had a higher breakdown viscosity than potato (2.195 Pa·s), wheat (0.775 Pa·s) and barley starch (1.938 Pa·s) (*1*, *24*, *25*). Therefore, the modification of native Bambara groundnut starch was necessary to improve its robustness. The low breakdown viscosity of the nanocomposite would render it stable at high processing temperatures due to its high thermal stability, making it a suitable ingredient for food products that are cooked at elevated temperatures and undergo extensive mixing in the preparation stage.

#### Setback viscosity

The setback viscosities of Bambara groundnut starch, soluble dietary fibre and nanocomposite were 2637, 122 and 128 mPa·s, respectively (Table 1). Both the groundnut soluble dietary fibre and the nanocomposite had significantly lower (p<0.001) setback viscosities than the starch. Setback viscosity refers to the tendency of starch to retrograde and undergo syneresis (*1*) and is an indication of gel stability (*18*). After gelatinisation, amylopectin recrystallises and transforms from an amorphous state to a more crystalline state causing the starch paste to thicken and form a stiff gel (*28*). When starch paste cools down, there is re-association between amylose and amylopectin molecules which results in the formation of a gel structure and consequently, increased viscosity (*21*, *26*). A high setback viscosity, as exhibited by Bambara groundnut starch, indicates a high tendency towards retrogradation and syneresis (*20*). Bambara groundnut soluble dietary fibre and nanocomposite are complex polymers with hydrogen and covalent bonds which inhibit the re-association of bonds following heating. The lower setback viscosity of the nanocomposite than of the starch could be attributed to the introduction of the soluble dietary fibre to the starch during manufacturing, resulting in the formation of new functional groups as shown by FTIR studies (*10*). These functional groups indicate the formation of new bonds which restrict the re-association of starch molecules after cooling. This further proved that Bambara groundnut soluble dietary fibre was successfully grafted onto the starch, reinforced the matrix and improved its characteristics. Gulu (*1*) reported the setback viscosity of Bambara groundnut starch of 1.957 Pa·s and established that inclusion complexes stabilise it. The differences in setback viscosities among studies could be attributed to the applied starch extraction methods, used machines and the variety of the tested Bambara groundnut.

#### Pasting temperature

When starch granules are exposed to elevated temperatures in the presence of water, they absorb water, swell and eventually rupture. The temperature at which they begin swelling is known as the pasting temperature (*27*). Pasting temperature gives an indication of the minimum temperature required to initiate starch gelatinisation (*26*). The pasting temperatures of Bambara groundnut starch (77.9 °C), its soluble dietary fibre (96.7 °C) and nanocomposite (87.4 °C) were significantly (p<0.001) different (Table 1). The results of the thermal properties of the starch were in fair agreement with its pasting properties where initial peak thermal degradation due to gelatinisation of native Bambara groundnut starch was reported to be 77.19 °C (*10*). Higher pasting temperatures of 84 and 83.2 °C for Bambara groundnut starch were reported by Adebowale *et al.* (*2*) and Afolabi (*22*), respectively. Pasting temperatures higher than those for the starch but lower than those for the nanocomposite and soluble dietary fibre were reported to be 82.0 and 82.2 °C for *Mucuna* bean starch (*2*) and barley starch (23), respectively. The pasting temperature of starch observed in this study is similar to that of Sirivongpaisal (*17*) and Gulu (*1*), who reported the pasting temperatures for Bambara groundnut starch of 77.7 and 79.90 °C, respectively.

Pasting temperatures of Bambara groundnut starch range between 77 and 84 °C, depending on the source and variety of seeds (*5*, *17*). The pasting temperature of the starch in this study was within this range, which is higher than that of potato starch (66.2−68.6 °C) and comparable to that of corn starch (77−88 °C) (*29*). The relatively high pasting temperature of pulses compared to cereal starches could be due to their high amylose contents (*30*). A higher pasting temperature is an indicator of thermal stability as more energy is required to rupture the granules. The higher pasting temperature of the nanocomposite was a confirmation of the presence of stronger bonds which required more energy to disrupt. The introduction of new functional groups as well as the disappearance and shifting of functional groups in the nanocomposite in FTIR studies confirmed the formation of a new, stronger complex (*10*).

#### Peak time

Peak time is the time when peak viscosity is reached (*31*). The peak times of Bambara groundnut starch, its soluble dietary fibre and the nanocomposite were 4.8, 7.0 and 6.9 min, respectively (Table 1). The peak times of all three biopolymers were significantly (p<0.001) different. A similar peak time of 4.70 min for the starch was reported by Gulu (*1*). Complexing increases the time required to reach peak viscosity (*1*) because more time is needed to break down the structure of the material under study. Hence, the nanocomposite biopolymer and its soluble dietary fibre (a complex polysaccharide) exhibited higher peak times than the starch (a simpler polysaccharide). A comparable peak time of 4.33 min for potato starch was reported by Kaur *et al.* (*24*). Potato starch is commonly used as a thickener in soups, sauces, custards and puddings (*32*), hence suggesting that Bambara groundnut starch could be employed for similar use in these systems.

Pasting studies showed that the pasting properties of Bambara groundnut starch would render it unsuitable for use as an ingredient in many food systems as it would breakdown at temperatures below 100 °C, gelatinise, retrograde and be prone to syneresis. Hence its modification by grafting it with its soluble dietary fibre to form a nanocomposite was justified. The nanocomposite has improved properties and would be useful as an ingredient in various food systems as it withstands high shear rates and temperatures. It exhibited a similar behaviour to some starches commonly used in the food industry, suggesting that it would behave in a similarly desirable manner.

### Bambara groundnut starch, soluble dietary fibre and its nanocomposite colour characteristics

The lightness (L*), redness/greenness (a*), yellowness/blueness (b*), chroma and hue angles of Bambara groundnut starch were 87.9, 1.3, 12.4, 12.5 and 83.9°, respectively, of the soluble dietary fibre 76.1, 2.2, 18.9, 19.0 and 83.3°, respectively, and of the nanocomposite 89.2, 0.4, 14.6, 14.6 and 88.42°, respectively (Table 2). The three biopolymers differed significantly (p<0.001) in all colour characteristics. The relatively higher degree of lightness observed of the nanocomposite can be attributed to the bleaching properties of H_2_O_2_ used in the formulation of the nanocomposite. All the polymers had positive a* and b* values, indicating that they were more associated with redness and yellowness, respectively.

**Table 2:** Colour attributes of Bambara groundnut starch (BGNS), Bambara groundnut soluble dietary fibre (BGN-SDF) and Bambara groundnut starch-fibre nanocomposite (STASOL)

**Sample**	L*	a*	b*	C*	***h°***	
**BGNS**	(87.9±0.0)^a^	(1.3±0.0)^a^	(12.4±0.0)^a^	(12.5±0.0)^a^	(83.9±0.0)^a^	
**BGN-SDF**	(76.1±0.0)^b^	(2.2±0.0)^b^	(18.9±0.0)^b^	(19.0±0.0)^b^	(83.3±0.0)^b^	
**STASOL**	(89.2±0.0)^c^	(0.4±0.0)^c^	(14.6±0.0)^c^	(14.6±0.0)^c^	(88.4±0.0)^c^	
**BGNS and BGN-SDF**						13.5
**BGNS and STASOL**						6.6
**BGN-SDF and STASOL**						14.2

Values are mean±standard deviation. Mean values followed by different superscripts within a column differ significantly (p≤0.05). L*=lightness, a*=red/ green, b*=yellow/blue

The redness and yellowness of the soluble dietary fibre are related to the phenolics and other antioxidant-possessing chemicals such as anthocyanins, cyanidins as well as a wide range of isoflavones and phenolic acids (*11*). The lightness of biopolymers destined for food use is of importance in the final products as it determines how much the original colour of the food system will be affected (*32*, *33*). As such, the nanocomposite can be used as an ingredient in food systems without negatively impacting their colour. It was observed that lightness increased with increasing hue and this was in agreement with Maphosa and Jideani (*4*), who studied the soluble dietary fibre of the Bambara groundnut black-eye variety and reported the colour attributes of L*=73.0, a*=1.7, b*=13.8, chroma=13.9 and hue angle=83.1°. These were comparable with the colour attributes reported in this study.

The colour differences among the biopolymers are presented in Table 2. A colour difference (*E*) of 1 is known as a just-noticeable difference and is the threshold at which trained observers notice the difference between two colours (*34*). The difference between two colours can be noticeable but still considered acceptable. A colour difference between 4 and 8 is acceptable. Above 8, the colour difference is deemed unacceptable and likely to be rejected by consumers (*35*). The colour differences between the Bambara groundnut starch and soluble dietary fibre, the starch and nanocomposite, and the soluble dietary fibre and nanocomposite were 13.5, 6.6 and 14.2, respectively. There was a noticeable difference in colour among the three biopolymers. The difference between the starch and nanocomposite (*E*=6.6) was considered acceptable as it falls in the 4−8 range (*4*), but other two colour differences were above 8 and unacceptable.

### Chemical composition of Bambara groundnut starch, soluble dietary fibre and starch-soluble dietary fibre nanocomposite

The chemical composition of Bambara groundnut starch, its soluble dietary fibre and nanocomposite is given in Table 3. Bambara groundnut starch and its soluble dietary fibre did not differ significantly in their moisture (p=0.620) and fat (p=0.116) mass fractions, while both had a significantly (p<0.001) higher moisture mass fraction and a significantly (p<0.001) lower fat mass fraction than the groundnut nanocomposite. All three biopolymers differed significantly (p<0.001) in energy, protein and carbohydrate composition. Bambara groundnut soluble dietary fibre and nanocomposite were not significantly (p=0.217) different in their ash mass fraction and both had a significantly (p<0.001) higher ash mass fraction than the starch.

**Table 3:** Proximate analysis of Bambara groundnut starch (BGNS), Bambara groundnut soluble dietary fibre (BGN-SDF) and Bambara groundnut starch-fibre nanocomposite (STASOL)

Sample	*w*/%					Energy/kJ
Moisture	Ash	Protein	Fat	Carbohydrates
BGNS	(10.8±0.0)^a^	(0.1±0.0)^a^	(1.7±0.4)^a^	(0.6±0.0)^a^	(86.8±0.3)^a^	(1525.8±0.7)^a^
BGN-SDF	(10.8±0.1)^a^	(4.9±0.1)^b^	(15.5±0.3)^b^	(0.5±0.0)^a^	(68.2±0.2)^b^	(1443±2)^b^
STASOL	(8.6±0.3)^b^	(4.9±0.1)^b^	(7.0±0.7)^c^	(0.8±0.0)^b^	(78.7±0.6)^c^	(1487±66)^c^

Values are mean±standard deviation. Mean values within a column followed by different letters in superscript differ significantly (p≤0.05)

**Fig. 1:**
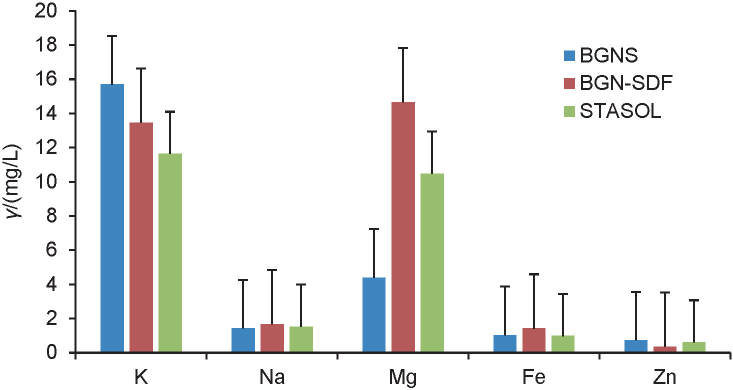
Mineral composition of Bambara groundnut starch, its soluble dietary fibre and nanocomposite

Ash is an indication of the mineral content of samples and the high values obtained in Bambara groundnut soluble dietary fibre and nanocomposite suggest that they may contribute micro- and macroelements to food systems (*17*, *34*). The moisture mass fraction of all three biopolymers in this study was similar to that of cassava starch (8.21−12.39%) reported by Agyepong and Barimah (*34*). Lower ash values (0.38-0.98%) were reported for modified cassava starch (*34*). These were considerably lower than those of the Bambara groundnut nanocomposite and this could be because the modification of the starch in the formation of the nanocomposite involved the introduction of a new biopolymer (Bambara groundnut soluble dietary fibre), which has a relatively high ash mass fraction (4.90%). Other starches are commonly modified using methods that involve the use of enzymes, heat and chemicals without the introduction of a second nutritional compound, hence the ash mass fraction is solely an indication of the minerals present in the starch. As such the nanocomposite makes a better fortifier of minerals in food products. The mineral composition of Bambara groundnut starch, its soluble dietary fibre and nanocomposite determined using inductively coupled plasma-optical emission spectrometry (ICP-OES) is presented in Fig 1.

Minerals are of importance in the physiological functioning of the human body. Bambara groundnut starch contained the highest mass concentration of potassium (15.69 mg/L) and zinc (0.72 mg/L) but the lowest mass concentration of sodium (1.42 mg/L) and magnesium (4.39 mg/L) among the samples (Fig. 1). Bambara groundnut soluble dietary fibre had the highest mass concentration of sodium (1.67 mg/L), magnesium (14.66 mg/L) and iron (1.41 mg/L). The nanocomposite had the lowest mass concentration of iron (0.98 mg/L) but a high mass concentration of potassium (11.64 mg/L) and magnesium (10.47 mg/L). The total mineral mass concentration of the biopolymers determined using ICP-OES agreed with the nutritional studies, with the soluble dietary fibre having the highest ash mass fraction and the starch the least (Table 3). Bambara groundnut soluble dietary fibre had the highest mass fraction of ash (4.90 g) and total mineral mass concentration (31.55 mg/L), while the starch had the lowest mass fraction of ash (0.07 g) and total mineral mass concentration (23.25 mg/L). This was because the minerals in nanocomposite were from the starch and soluble dietary fibre used in the production. Generally, the values for most of the minerals in this study were lower than those reported for Bambara groundnut seeds (*2*, *36*). This was expected because only the components of Bambara groundnut were analysed in this study, while whole seeds were reported by other researchers.

### Hydration and oil-binding properties of Bambara groundnut starch, its soluble dietary fibre and nanocomposite

The solubility index, water absorption capacity (WAC) and oil-binding capacity (OBC) of Bambara groundnut starch, its soluble dietary fibre and nanocomposite are given in Table 4.

**Table 4:** Physicochemical properties of Bambara groundnut starch (BGNS), Bambara groundnut soluble dietary fibre (BGN-SDF) and Bambara groundnut starch-fibre nanocomposite (STASOL)

**Sample**	**Water absorption capacity/(g/g)**	**Solubility index/%**	**Oil-binding capacity/(g/g)**	**EAI/%**	**ESI/%**
**BGNS**	(1.6±0.2)^a^	(0.9±0.2)^a^	(1.1±0.1)^a^	(23.2±32.0)^a^	(23.3±18.2)^a^
**BGN-SDF**	-	(95.3±0.0)^b^	(3.8±0.1)^b^	(85.7±6.0)^b^	(87.1±6.5)^b^
**STASOL**	-	(93.3±0.1)^c^	(1.5±0.1)^c^	(90.6±1.9)^c^	(87.5±3.3)^c^

Values are mean±standard deviation. Mean values within a column followed by a different letter in superscript differ significantly (p≤0.05) EAI=emulsifying activity index, ESI=emulsion stability index

Solubility is defined by the IUPAC (*37*) as the analytical composition of a saturated solution expressed as a proportion of a designated solute in a designated solvent. A high solubility index positively affects the functional properties both in food systems and in the gastrointestinal canal (*37*). All biopolymers showed significant (p<0.001) differences in the solubility index with native Bambara groundnut starch being practically insoluble in water (0.9%) (Table 4). Native starch is insoluble in cold water because the amylose and amylopectin chains are arranged in a tight interlocking structure that becomes a hydrophobic crystalline-like starch granule (*1*). This was in agreement with the results of Agyepong and Barimah (*34*), who reported very low solubility of native cassava starch in the range of 1.29−7.38%. Furthermore, they reported an increase in the solubility of starch after modification. This was also observed for the nanocomposite in this study. The smaller average particle size (74.01 nm) of the nanocomposite (*10*) means that it has a large surface area, hence increasing its interaction with water molecules. The high solubility of the nanocomposite makes it a useful ingredient in food preparations such as soups, dairy products, beverages, coffee creamers and gravies (*38*).

WAC is the ability of a sample to retain water, which plays a significant role in food systems where the sample is used as a bulking agent, stabiliser, thickener and anticaking agent (*2*). The WAC of Bambara groundnut starch was 1.6 g/g, while that of its soluble dietary fibre and nanocomposite could not be determined as they were significantly soluble in water, as shown in Table 4. A similar WAC of the starch was reported by Sirivongpaisal (*17*) (1.67 g/g), while Gulu (*1*) reported a lower value (1.17 g/g). Differences could be attributed to the different Bambara groundnut varieties. Gulu (*1*) used mixed Bambara groundnut seeds, while only the black-eye variety was used here in the production of the nanocomposite. Adebowale *et al.* (*2*) reported a higher WAC (2.0 g/g) for the starch, which could be attributed to the different extraction methods. Adebowale *et al.* (*2*) employed a wet milling method, while a dry milling method was used in this study. The relatively lower WAC reported in this study suggested that the starch could find use in food where moderate swelling is required (*35*).

The OBC of Bambara groundnut starch, its soluble dietary fibre and nanocomposite differed significantly (p<0.001) and was 1.1, 3.8 and 1.5 g/g, respectively (Table 4). This is the ability of lipids to form interactions with non-polar side chains of molecules (*35*) and is affected by factors such as particle size, surface area, charge and hydrophobicity (*2*). It works on the principle of physically entrapping oil through capillary attraction (*1*). It indicates the ability and extent to which biopolymers can stabilise high-fat food products and emulsions as well as act as fat binders and replacers in products such as meat (*39*). The OBC of native starch in this study (1.1 g/g) was lower than its WAC (1.6 g/g) (Table 4), which indicated a higher level of hydrophilicity than of hydrophobicity. The low OBC of the starch than of the nanocomposite and soluble dietary fibre could be attributed to the low levels of proteins (Table 3). The proteins present in the nanocomposite and soluble dietary fibre may be hydrophobic, hence would display superior lipid-binding characteristics (*38*). The ability of biopolymers and biopolymer nanocomposites like the one used here to bind oil can be applied in the food industry to reduce fat loss during cooking as well as in foodstuffs such as meat, emulsions and baked goods, thus improving palatability, flavour retention and shelf life (*40*). Physiologically, the OBC of biopolymers allows them to play a significant role in bile acid absorption and consequently cholesterol reduction (*35*).

### Emulsion activity and stability indices of Bambara groundnut starch, its soluble dietary fibre and nanocomposite

Emulsifying activity index (EAI) is the ability of a compound to participate in the formation and stabilisation of a newly created emulsion (*41*), while emulsion stability index (ESI) measures the ability of an emulsion to resist changes in its structure and maintain its physicochemical properties over time (*35*). The significantly (p<0.001) lower EAI and ESI of Bambara groundnut starch can be highly attributed to its insolubility and low WAC (Table 4). Biopolymers find use as stabilisers in food emulsions. They accomplish this function by increasing the viscosity of the system, thereby retarding oil droplet migration (*42*). The EAI and ESI of the nanocomposite and soluble dietary starch suggest that they would effectively carry out these functions. To successfully increase the viscosity of a system, a polysaccharide needs to be adequately soluble in the aqueous phase. The low hydration properties of the starch, therefore, hindered it from being a suitable emulsion stabiliser. As emulsions have a lipid component, a suitable stabiliser needs a suitable degree of hydrophobicity to adequately perform a stabilising function (*43*). The soluble dietary fibre and nanocomposite both exhibited significantly (p≤0.05) higher hydration and OBC (Table 4), thus making them good potential emulsion stabilisers.

### Mass fraction of glucose in Bambara groundnut starch, soluble dietary fibre and starch-soluble dietary fibre nanocomposite

Table 5 shows the glucose mass fraction in Bambara groundnut starch, its soluble dietary fibre and nanocomposite. In Table 3, the carbohydrate mass fraction in the starch was 86.8%. This was in fair agreement with the results of this section. Starch is made up of repeating units of glucose (*44*), hence its hydrolysis yielded 87.9% glucose, similar to its carbohydrate mass fraction.

**Table 5:** Antioxidant properties and glucose content of Bambara groundnut starch (BGNS), Bambara groundnut soluble dietary fibre (BGN-SDF) and Bambara groundnut starch-fibre nanocomposite (STASOL)

**Sample**	**Polyphenols as *w*(GAE)/(mg/g)**	**FRAP as *b*(AAE)/(μmol/g)**	***w*(glucose)/%**
**BGNS**	(0.10±0.01)^a^	(1.2±0.4)^a^	87.9
**BGN-SDF**	(6.6±0.2)^b^	(4.8±0.8)^b^	N.D.
**STASOL**	(0.46±0.05)^c^	(1.4±0.2)^c^	63.4

Values are mean±standard deviation. Mean values within a column followed by different letters in superscript are significantly different (p≤0.05). FRAP=ferric reducing antioxidant power, GAE=gallic acid equivalents, AAE=ascorbic acid equivalents, N.D.=not detected

The nanocomposite had a higher carbohydrate mass fraction (78.7%), as shown in Table 3, than its glucose mass fraction (63.4%) (Table 5). Proportionately, its glucose mass fraction should have been higher, however, some may have been lost during production. The nanocomposite is made of Bambara groundnut starch to its soluble dietary fibre ratio 15:1.95, hence the quantified glucose most probably originates from the hydrolysis of the starch and the difference would have been carbohydrates from the soluble dietary fibre, which is not made up of glucose units. Glucose was not detected in the soluble dietary fibre. This was in agreement with Maphosa and Jideani (*4*), who reported an insignificant amount of glucose (<1%) in the Bambara groundnut soluble dietary fibre.

### Antioxidant properties of Bambara groundnut soluble dietary fibre, its soluble dietary fibre and nanocomposite

The content of total polyphenolic compounds (TPC) and ferric reducing antioxidant power (FRAP) value of biopolymers suggest that they have antioxidant properties. The TPC and FRAP values of the tested biopolymers in Table 5 show that they differed significantly (p<0.001). The soluble dietary fibre is rich in antioxidant compounds, while starch is generally low in active compounds (*4*). This validated the results obtained in this study, whereby the soluble dietary fibre had the highest amount of polyphenolic and ferric reducing compounds, while the starch had significantly (p<0.001) low antioxidant capability. This proved that antioxidant compounds were successfully delivered from the soluble dietary fibre to the starch. However, since a low percentage (11.5%) of the soluble dietary fibre was incorporated into the nanocomposite, its total antioxidant activity was lower than of the soluble dietary fibre.

Jayawardena *et al.* (*45*) reported the total phenolic mass fraction of 22 fruit juices, expressed as GAE, in the range of 0.24−0.39 mg/g. These could be a result of the presence of common antioxidants such as vitamin C, especially in the citrus juices. These results were lower than those reported for the nanocomposite (0.46 mg/g). Fruit juices are a reliable source of antioxidants; therefore the superior antioxidant property of the nanocomposite is a positive characteristic.

The significantly (p<0.001) higher phenolic content of the nanocomposite than of the starch could also be attributed to the chemical modification used in the production of the nanocomposite. To expose the reactive starch functional groups, ascorbic acid was oxidised by hydrogen peroxide, resulting in the formation of hydroxyl and ascorbate radical intermediates (*46*). These intermediates may have interfered with the hydrogen atom transfer mechanism of oxygen radical absorbance capacity (ORAC) assay, thereby influencing the antioxidant mechanism and leading to the higher antioxidant capacity of the nanocomposite (*1*).

The FRAP, expressed as AAE, of Bambara groundnut starch, it soluble dietary fibre and nanocomposite was 1.2, 4.8 and 1.4 μmol/g, respectively (Table 5). All three biopolymers differed significantly (p<0.001) in their Fe(III) reducing capabilities. The FRAP assay is based on the reduction of Fe(III) ions to Fe(II) ions by the tested compound and is often used to measure the antioxidant capacity of foods and beverages containing polyphenols (*47*). The polyphenolic content of the nanocomposite suggests that it can be exploited as a novel antioxidant and would be of importance in protecting against superoxide radicals, free radicals and lipid peroxidation (*39*). It would therefore find use in fatty food products to improve oxidative stability, thereby extending their shelf life (*39*). As a natural antioxidant, it would be a suitable alternative to artificial antioxidants, which have been reported to be carcinogenic and teratogenic, hence their use in food products is discouraged (*48*).

### Polyphenolic compounds in Bambara groundnut starch, soluble dietary fibre and starch-soluble dietary fibre nanocomposite

Table 6 shows the phenolic composition of the tested biopolymers. All three biopolymers contained appreciable mass fractions of phenolics. The phenolic compounds chlorogenic acid (18 mg/g), monocrotaline (20 mg/g), luteolin 7-O-(6''-malonylglucoside) (4 mg/g) and casuarine 6-α-d-glucoside (27 mg/g) were present in the soluble dietary fibre but absent from the starch and nanocomposite, while blumealactone C (7 mg/g) was present in the starch but absent from the other two biopolymers. Of the three biopolymers, the soluble dietary fibre had the highest mass fraction of phenolics compared to the starch and nanocomposite, with high mass fractions of (+)-sesamin (12 mg/g), 12,13-TriHOME (8602 mg/g), dronabinol (3389 mg/g), 9(S)-HPODE (50 mg/g), α-dimorphecolic acid (273 mg/g), dimethyltryptamine (7281 mg/g) and [1R-(1α,4aβ,6α,8aα)]-1,2,4a,5,6,8a-hexahydro-6-hydroxy-4,7-dimethyl-a-methylene-1-naphthaleneacetic acid methyl ester (7594 mg/g).

**Table 6:** Phenolic composition of Bambara groundnut starch (BGNS), Bambara groundnut soluble dietary fibre (BGN-SDF) and Bambara groundnut starch-fibre nanocomposite (STASOL)

Phenolic compound	Phenolic group	*t*_elution_/min		*w*(phenolic compound)/(mg/g)	
			BGNS	BGN-SDF	STASOL
Scoparone	Flavonoid	7.19	0	11	28
Chlorogenic acid	Phenol esters	7.60	0	18	0
Blumealactone C	Terpene lactone	7.71	7	0	0
(+)-Sesamin	Polyphenol	8.12	4	12	11
4-hydroxymethyl-2-methoxyphenyl-1-O-β-d-apiofuranosyl-(1->6)-O-β-d-glucopyranoside	Flavonone	8.40	0	10	6
Furcatin	Phenylpropene	8.72	4	6	0
Monocrotaline	Pyrrolizidine alkaloid	9.30	0	20	0
[1R-(1α,4aβ,6α,8aα)]-1,2,4a,5,6,8a-hexahydro-6-hydroxy-4,7-dimethyl-a-methylene-1-naphthaleneacetic acid methyl ester	Sesquiterpenoid	9.99	237	7594	8
Luteolin 7-O-(6''-malonylglucoside)	Flavonoid	10.23	0	4	0
Dimethyltryptamine	Phenol amide	10.48	1446	7281	13
Isatidine	Pyrrolizidine alkaloid	10.63	8	27	0
Casuarine 6-α-d-glucoside	Pyrrolizidine alkaloid	10.95	0	27	0
9,12,13-TriHOME	Linoleic acid derivative	12.92	396	8602	3009
Dronabinol	Tetrahydrocannabinol	13.94	103	3389	643
9(S)-HPODE	Linoleic acid derivative	14.12	26	50	63
α-dimorphecolic acid	Linoleic acid derivative	14.35	49	273	161

The polyphenolic compounds reported in this section were in agreement with the TPC and FRAP results (Table 5), where the soluble dietary fibre had the highest mass fraction of total polyphenols and the strongest ferric acid-reducing power. Starch generally has low antioxidant activity (*4*) and this was demonstrated by its relatively lower phenolic mass fraction (Table 6). The presence of phenolic compounds in the nanocomposite demonstrated that active compounds were successfully delivered from the soluble dietary fibre to starch. Therefore, it was concluded that the nanocomposite contained appreciable mass fractions of antioxidant compounds. However, the phenolic compounds furcatin, dimethyltryptamine, isatidine, casuarine and 6-α-d-glucoside [1R-(1α,4aβ,6α,8aα)]-1,2,4a,5,6,8a-hexahydro-6-hydroxy-4,7-dimethyl-a-methylene-1-naphthaleneacetic acid methyl ester, luteolin 7-O-(6''-malonylglucoside) were present in high mass fractions in the starch and soluble dietary fibre but were either not delivered at all to the nanocomposite or were delivered in very low mass fractions. This was attributed to the low amount of soluble dietary fibre used in the formulation of nanocomposite. As such, future studies should look into using a higher amount of soluble dietary fibre to increase the antioxidant capacity of the nanocomposite.

The findings of this study highlight the potential of Bambara groundnut as an economic source of natural antioxidants for human consumption and could therefore open horizons to its industrial use in the development of functional food. The antioxidant activity of its seeds and constituents has been reported by several researchers (*5*, *11*). The antioxidant properties of phenolics arise from the ability of their hydroxyl groups to donate hydrogen as well as react with oxygen and nitrogen species, producing radical species in a termination reaction (*49*). Phenolic compounds such as those identified in these biopolymers have been reported to possess many desirable characteristics such as anti-inflammatory, antimicrobial, antifungal, antiallergic, antioxidant, antiapoptotic, antitumour and estrogenic activity (*50*).

## CONCLUSIONS

The grafting of Bambara groundnut soluble dietary fibre onto the groundnut starch successfully mitigated the undesirable characteristics of both biopolymers in their native state while retaining their desirable characteristics. Bambara groundnut nanocomposite possesses desirable properties making it a suitable ingredient in various food products. The physicochemical, antioxidant and functional properties of the nanocomposite make it a desirable emulsion stabilizer, thickening agent and fortifier for delivering active compounds, such as phenolics, to food systems. Unlike the Bambara groundnut starch, the nanocomposite did not exhibit typical pasting properties, thus confirming the formation of a new compound. Its light colour allows its use as an ingredient without a negative impact on the colour of food systems. Biopolymers like Bambara groundnut starch-soluble dietary fibre nanocomposite have the potential to revolutionise the food industry.

## References

[1] Gulu NB. Functional and rheological properties of Bambara groundnut starch-catechin complex obtained by chemical grafting [MSc Thesis]. Cape Town, South Africa: Cape Peninsula University of Technology; 2018.

[2] Adebowale KO Afolabi T Lawal OS. Isolation, chemical modification and physicochemical characterisation of Bambarra groundnut (*Voandzeia subterranea*) starch and flour. Food Chem. 2002;78(3):305–11. 10.1016/S0308-8146(02)00100-0

[3] Singh N Kaur L. Morphological, thermal, rheological and retrogradation properties of potato starch fractions varying in granule size. J Sci Food Agric. 2004;84(10):1241–52. 10.1002/jsfa.1746

[4] Maphosa Y Jideani VA. Physicochemical characteristics of Bambara groundnut dietary fibres extracted using wet milling. S Afr J Sci. 2016;112(1/2):1–8. 10.17159/sajs.2016/20150126

[5] Oyeyinka SA Singha S Adebola P Amonsou E. Physicochemical properties of starches with variable amylose contents extracted from Bambara groundnut genotypes. Carbohydr Polym. 2015;133:171–8. 10.1016/j.carbpol.2015.06.100 26344269

[6] Park EY Kim HN Kim JY Lim ST. Pasting properties of potato starch and waxy maize starch mixtures. Starch. 2009;61(6):352–7. 10.1002/star.200800029

[7] Lin JH Kao WT Tsai YC Chang YH. Effect of granular characteristics on pasting properties of starch blends. Carbohydr Polym. 2013;98(2):1553–60. 10.1016/j.carbpol.2013.07.039 24053839

[8] Novelo-Cen L Betancur-Ancona D. Chemical and functional properties of *Phaseolus lunatus* and *Manihot esculenta* starch blends. Starch. 2005;57(9):431–41. 10.1002/star.200500398

[9] Zhang J Gao Y Qian S Liu X Zu H. Physicochemical and pharmacokinetic characterization of a spray-dried malotilate emulsion. Int J Pharm. 2011;414(1-2):186–92. 10.1016/j.ijpharm.2011.05.032 21619915

[10] Maphosa Y Jideani VA Ikhu-Omoregbe DI. *Vigna* *subterranea* (L.) Verdc starch-soluble dietary fibre potential nanocomposite: Thermal behaviour, morphology and crystallinity. Processes (Basel). 2022;10(2):299. 10.3390/pr10020299

[11] Murevanhema YY Jideani V. Potential of Bambara groundnut (*Vigna subterranea* (L.) Verdc) milk as a probiotic beverage: A review. Crit Rev Food Sci Nutr. 2013;53(9):954–67. 10.1080/10408398.2011.574803 23768187

[12] Sharma A. Understanding color management. Clifton Park, NY, USA: Thomson/Delmar Learning; 2005. pp. 16-21.

[13] Official Methods AOAC. Rockville, MD, USA: AOAC International; 2011.

[14] Lai P Shiau CJ Wang CC. Effects of oligosaccharides on phase transition temperatures and rheological characteristics of waxy rice starch dispersion. J Sci Food Agric. 2012;92(7):1389–94. 10.1002/jsfa.4712 22083791

[15] Chove B Grandison A Lewis M. Emulsifying properties of soy protein isolates obtained by isoelectric precipitation. J Sci Food Agric. 2001;81(8):759–63. 10.1002/jsfa.877

[16] Statistical IBM. Package for the Social Science (SPSS), Statistics for Windows, v. 22.0, IBM Corp., Armonk, NY, USA; 2013. Available from: https://www.ibm.com/products/spss-statistics.

[17] Sirivongpaisal P. Structure and functional properties of starch and flour from bambarra groundnut. Songklanakarin J Sci Technol. 2008;30 Suppl 1:51–6.

[18] Mensah NG. Modification of Bambara groundnuts starch, composited with defatted Bambara groundnut flour for noodle formulation [MSc Thesis]. Kumasi, Ghana: Kwame Nkrumah University of Science and Technology, Kumasi College of Science; 2011.

[19] Ragaeea S Abdel-Aal EM. Pasting properties of starch and protein in selected cereals and quality of their food products. Food Chem. 2006;95(1):9–18. 10.1016/j.foodchem.2004.12.012

[20] Amoo ARN Wireko-Manu FD Oduro I. Physicochemical and pasting properties of starch extracted from four yam varieties. Int J Food Sci Nutr. 2014;2(6):262–9. 10.11648/j.jfns.20140206.14

[21] Falade KO Semon M Fadairo OS Oladunjoye AO Orou KK. Functional and physico-chemical properties of flours and starches of African rice cultivars. Food Hydrocoll. 2014;39:41–50. 10.1016/j.foodhyd.2013.11.002

[22] Afolabi TA. Synthesis and physicochemical properties of carboxymethylated bambara groundnut (*Voandzeia subterranea*) starch. Int J Food Sci Technol. 2012;47(3):445–51. 10.1111/j.1365-2621.2011.02860.x

[23] Gujral HS Sharma P Kaur H Singh J. Physiochemical, pasting, and thermal properties of starch isolated from different barley cultivars. Int J Food Prop. 2011;16(7):1494–506. 10.1080/10942912.2011.595863

[24] Kaur A Singh N Ezekiel R Guraya HS. Physicochemical, thermal and pasting properties of starches separated from different potato cultivars grown at different locations. Food Chem. 2007;101(2):643–51. 10.1016/j.foodchem.2006.01.054

[25] Fan X Zhu J Dong W Sun Y Lv C Guo B. Comparison of pasting properties measured from the whole grain flour and extracted starch in barley (*Hordeum vulgare* L.). PLoS One. 2019;14(5):e0216978. 10.1371/journal.pone.0216978 31141562 PMC6541268

[26] Jan R Sharma S Saxena DC. Pasting and thermal properties of starch extracted from *Chenopodium album* grain. Int J Res Agric Food Sci. 2013;4(10):981–8.

[27] Araujo-Farro PC, do Amaral Sobral JP, Menegalli FC. Comparison of starch pasting and retrogradation properties of quinoa (*Chenopodium quinoa* Willd), rice, potato, cassava, wheat and corn starches. Proceedings of the 2nd Mercosur Congress on Chemical Engineering and 4th Mercosur Congress on Process Systems Engineering; 2005 August 14-18, Rio de Janeiro, Brazil; 2005. pp. 1-7.

[28] Cornejo-Ramírez YI Martínez-Cruz O Del Toro-Sánchez CL Wong-Corral FJ Borboa-Flores J Cinco-Moroyoqui FJ. The structural characteristics of starches and their functional properties. CYTA J Food. 2018;16(1):1003–17. 10.1080/19476337.2018.1518343

[29] Joshi M Aldred P Mcknight S Panozzo J. Physicochemical and functional characteristics of lentil starch. Carbohydr Polym. 2013;92(2):1484–96. 10.1016/j.carbpol.2012.10.035 23399180

[30] Hoover R Hughes T Chung HJ Liu Q. Composition, molecular structure, properties, and modification of pulse starches: A review. Food Res Int. 2010;43(2):399–413. 10.1016/j.foodres.2009.09.001

[31] Griess JK Mason SC Jackson DS Galusha TD Pedersen JF Yaseen M. Environment and hybrid influences on rapid-visco-analysis flour properties of food-grade grain sorghum. Crop Sci. 2011;51(4):1757–66. 10.2135/cropsci2010.10.0604

[32] Jane J Chen YY Lee LF McPherson AE Wong KS Radosavljevic M Effects of amylopectin branch chain length and amylose content on the gelatinization and pasting properties of starch. Cereal Chem. 1999;76(5):629–37. 10.1094/CCHEM.1999.76.5.629

[33] Tosh SM Yada S. Dietary fibres in pulse seeds and fractions: Characterisation, functional attributes and applications. Food Res Int. 2010;43(2):450–60. 10.1016/j.foodres.2009.09.005

[34] Agyepong JK Barimah J. Physicochemical properties of starches extracted from local cassava varieties with the aid of crude pectolytic enzymes from *Saccharomyces cerevisiae* (ATCC 52712). Afr J Food Sci. 2018;12(7):151–64. 10.5897/AJFS2018.1701

[35] Eltayeb ARSM Ali O Abou-Arab AA Abu-Salem FM. Chemical composition and functional properties of flour and protein isolate extracted from Bambara groundnut (*Vigna subterranean*). Afr J Food Sci. 2011;5(2):82–90.

[36] Olayele AA Adeyeye EI Adesina AJ. Chemical composition of Bambara groundnut (*V. subterranea* L. Verdc) seed parts. Bangladesh J Sci Ind Res. 2013;48(3):167–78. 10.3329/bjsir.v48i3.17325

[37] McNaught AD, Wilkinson A, editors. Compendium of chemical terminology, The gold book. Research Triangle Park, NC, USA: International Union of Pure and Applied Chemistry (IUPAC); 2006.

[38] Adeleke OR Adiamo OQ Fawale OS. Nutritional, physicochemical, and functional properties of protein concentrate and isolate of newly-developed Bambara groundnut (*Vigna subterrenea* L.) cultivars. Food Sci Nutr. 2018;6(1):229–42. 10.1002/fsn3.552 29387383 PMC5778210

[39] Elleuch M Bedigian D Roiseux O Besbes S Blecker C. Dietary fibre and fibre-rich by-products of food processing; Characterisation technological functionality and commercial applications: A review. Food Chem. 2011;124(2):411–21. 10.1016/j.foodchem.2010.06.077

[40] Slavin J. Fibre and prebiotics: Mechanisms and health benefits. Nutrients. 2013;5(4):1417–35. 10.3390/nu5041417 23609775 PMC3705355

[41] Mubaiwa J Fogliano V Chidewe C Linnemann AR. Bambara groundnut (*Vigna subterranea* (L.) Verdc.) flour: A functional ingredient to favour the use of an unexploited sustainable protein source. PLoS ONE. 2018;13(10):e0205776. 10.1371/journal.pone.0205776 30321223 PMC6188868

[42] Dickinson E. Interfacial structure and stability of food emulsions as affected by protein-polysaccharide interactions. Soft Matter. 2008;4(5):932–42. 10.1039/b718319d 32907124

[43] Cheong KW Tan CP Mirhosseini H Joanne-Kam WY Hamid NSA Basri M. The effect of prime emulsion components as a function of headspace concentration of soursop flavor compounds. Chem Cent J. 2014;8:23. 10.1186/1752-153X-8-23 24708894 PMC3997474

[44] Tayade R Kulkarni KP Jo H Song JT Lee JD. Insight into the prospects for the improvement of seed starch in legume- A review. Front Plant Sci. 2019;10:1213. 10.3389/fpls.2019.01213 31736985 PMC6836628

[45] Jayawardena N Watawana MI Waisundara VY. The total antioxidant capacity, total phenolics content and starch hydrolase inhibitory activity of fruit juices following pepsin (gastric) and pancreatin (duodenal) digestion. J Verbr Lebensm. 2015;10:349–57. 10.1007/s00003-015-0951-y

[46] Spizzirri UG Parisi OI Iemma F Cirillo G Puoci F Curcio M Antioxidant-polysaccharide conjugates for food application by eco-friendly grafting. Carbohydr Polym. 2009;79(2):333–40. 10.1016/j.carbpol.2009.08.010

[47] Marc G Stana A Oniga SD Pîrnău A Vlase L Oniga O. New phenolic derivatives of thiazolidine-2,4-dione with antioxidant and antiradical properties: Synthesis, characterization, *in vitro* evaluation, and quantum studies. Molecules. 2019;24(11):2060. 10.3390/molecules24112060 31151176 PMC6600258

[48] Betancur-Ancona D Perza-Mercado G Moguel-Ordonez Y Fuertes-Blanco S. Physicochemical characterisation of lima beans (*Phaseolus lunatus*) and jack bean (*Canavalia ensiformis*) fibrous residues. Food Chem. 2004;84(2):287–95. 10.1016/S0308-8146(03)00213-9

[49] Valentão P Fernandes E Carvalho F Andrade PB Seabra RM Bastos ML. Studies on the antioxidant activity of *Hypericum androsaemum* infusion: Scavenging activity against superoxide radical, hydroxyl radical and hypochlorus acid. Biol Pharm Bull. 2002;25(10):1320–3. 10.1248/bpb.25.1320 12392087

[50] Gagliardini E, Benigni A, Perico N. Pharmacological induction of kidney regeneration. In: Orlando G, Remuzzi G, Williams D, editors. Kidney transplantation, bioengineering and regeneration. Kidney transplantation in the regenerative medicine era. London, UK: Academic Press; 2017. pp. 1025-37. 10.1016/B978-0-12-801734-0.00074-6 10.1016/B978-0-12-801734-0.00074-6

